# Efficacy and experience of arthroscopic lateral patella retinaculum releasing through/outside synovial membrane for the treatment of lateral patellar compression syndrome

**DOI:** 10.1186/s12891-020-3130-y

**Published:** 2020-02-17

**Authors:** Ji-Bin Chen, Dong Chen, Ya-Ping Xiao, Jian-Zhong Chang, Te Li

**Affiliations:** 1 Department of Orthopedics, Wuhan Hanyang Hospital, Wuhan University of Science and Technology, Wuhan, 430050 China; 2 Department of Orthopedic Surgery, CR & WISCO General Hospital, Wuhan University of Science and Technology, Wuhan, China; 3 Department of Orthopedic Surgery, General Hospital of Central Theater Command, 68 Huangpu Road, Jiangan District, Wuhan, China

**Keywords:** Arthroscopy, Lateral patellar compression syndrome, Lateral patella retinaculum releasing, Synovial membrane

## Abstract

**Background:**

Arthroscopic closure release includes arthroscopic lateral patella retinaculum releasing (LPRR) either outside synovial membrane (OSM) or through synovial membrane (TSM). At present, there is no research to compare the clinical efficacy of the above two methods for the treatment of lateral patellar compression syndrome (LPCS). So, the goal of this study was to investigate the method and overcome of arthroscopic LPRR either OSM or TSM for the treatment of LPCS.

**Methods:**

From September 2014 to December 2017, 125 patients of LPCS underwent arthroscopic LPRR either OSM or TSM combined with joint debridement. In the OSM group, knee joint was cleaned first. The surface of lateral patella retinaculum (LPR) was created the chamber for arthroscopic operation to release LPR. Synovial membrane was retained. In the TSM group, knee joint was cleaned first. Then synovial membrane, joint capsule and LPR, and superficial fascia were gradually incised from the joint cavity to subcutaneous tissue. The synovial membrane was cut open. Before and after surgery, Lysholm score, patella medial shift, Kujala score, VAS score and surgical complications were collected for evaluating clinical overcomes.

**Results:**

All patients were followed up for 1.5–5 years. All patients had significant reduction in knee pain and improved function after 1 month and 1 year. The Lysholm score, the distance of patella medial shift, Kujala score, and VAS score in the OSM group and the TSM group were significantly improved in the final follow-up compared with before surgery (All *P* < 0.001), but these observed targets before surgery and at the last follow-up were compared between the OSM group and the TSM group with no statistical differences. However, the number of occurrences of joint hematoma and adhesion was significantly higher in the TSM group than the OSM group (*P* = 0.024).

**Conclusions:**

Arthroscopic closing LPRR for the treatment of LPCS can effectively improve the function and symptoms of patellofemoral joint with the advantages of small trauma, rapid recovery and less complications. But, the number of occurrences of hemarthrosis and joint adhesion were significantly higher in the TSM group than in the OSM group.

**Trial registration:**

The trial registration number (IRCT): IRCT20200205046378N1 and date of registration: February 10, 2020 (retrospectively registered).

## Background

Lateral patellar compression syndrome (LPCS) is a disease of musculoskeletal disorder with pathological manifestations of the increased lateral patellofemoral joint pressure, which is caused by the long-term lateral tilt of patella, the adaptive tightening of lateral patella retinaculum (LPR), and the long-term stress imbalance of medial and lateral articular surfaces. The main clinical manifestations are patellofemoral joint pain, abnormal patella trajectory and articular cartilage injury [1, 2]. LPCS has become one of the main causes of anterior knee pain [1]. At present, the prevalence of anterior knee pain in the whole population is as high as 8.5–17.0%, but women are obviously higher than men [3, 4].

Many studies have confirmed the effectiveness of lateral patella retinaculum releasing (LPRR), extension of LPR, and lateral patelloplasty [2, 5, 6]. However, the gold standard of correction surgery for LPCS currently has not been determined. In 1974, Merchant et al. [7] proposed the LPRR for the treatment of LPCS. Nowadays, LPRR was the most widely used in clinical practice. The methods of the release include open incision, arthroscopic assisted incision and arthroscopic closure [8–10]. Among them, the most widely used method is arthroscopic closing release.

Arthroscopic closure release includes arthroscopic LPRR either outside synovial membrane (OSM) or through synovial membrane (TSM). At present, there is no research to compare the clinical efficacy of the above two methods. So, the goal of this study was to investigate the method and efficacy of arthroscopic LPRR either OSM or TSM for the treatment of LPCS and compared the overcomes between arthroscopic LPRR OSM and TSM to find out which of the two methods is superior. Before and after surgery, Lysholm score, patella medial shift, Kujala score, VAS score and surgical complications were collected for evaluating clinical overcomes.

## Methods

**Fig. 1:**
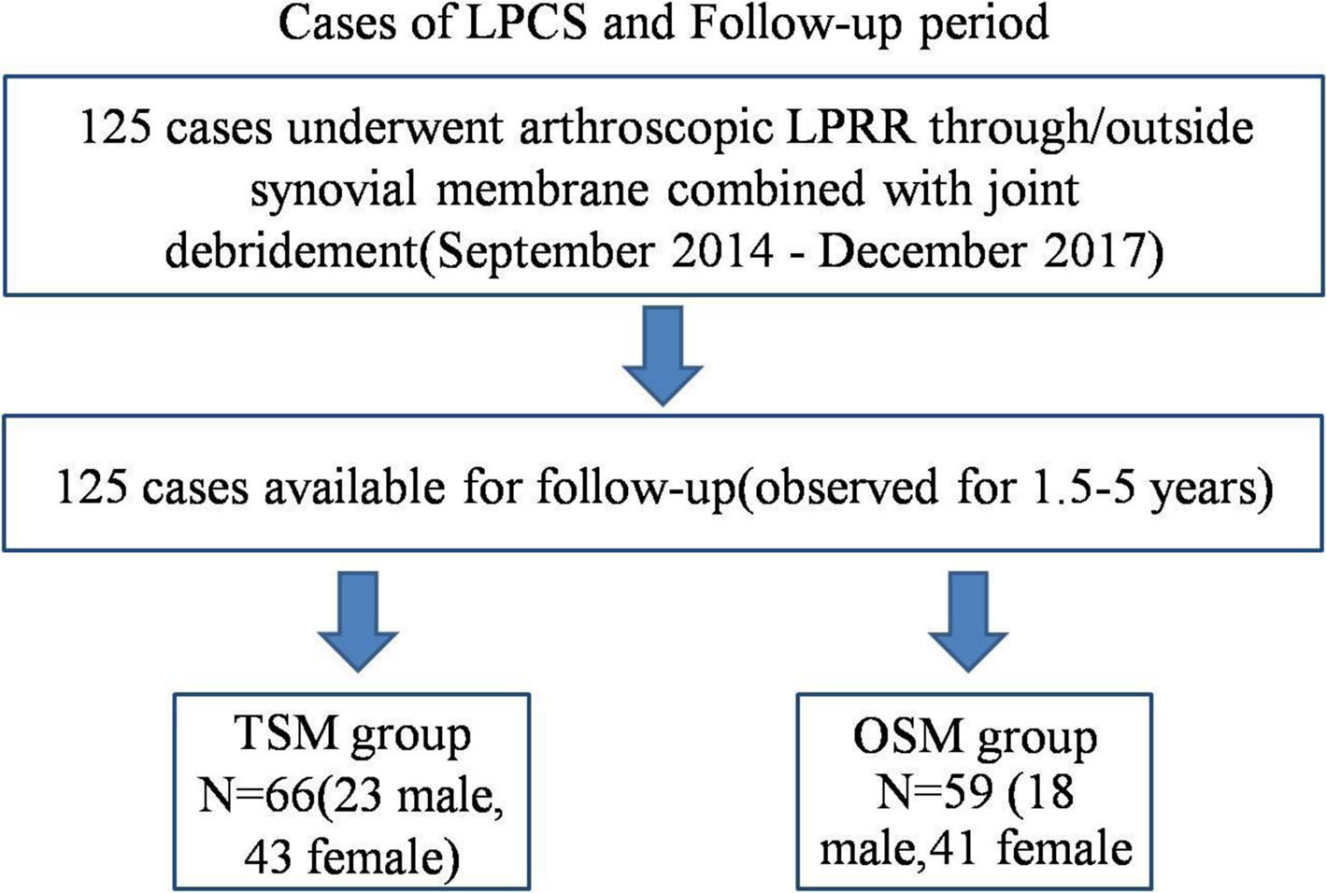
Cases of LPCS and Follow-up period

This study was a prospective study. The authors performed arthroscopic LPRR OSM or TSM combined with joint debridement in 125 patients of LPCS from September 2014 to December 2017 (Fig. 1). The method of simple random sampling was used to randomly divide the cases into the arthroscopic LPRR OSM group (OSM group) and TSM group (TSM group). Data collectors were blinded to the treatment assignment when collecting any of the outcomes. This study was approved by the institutional review board (IRB) of the authors’ hospitals, and informed consent was obtained from all patients.

### Inclusion and exclusion criteria

Inclusion criteria were as follows: 1 The symptoms of anterior knee pain in the unilateral knee joint were not significantly relieved after 3–6 months of standardized non-surgical treatment; 2 Preoperative examination showed that the LPR was tightened; 3 Imaging examination prompted the patella tilt to the outside and Q angle was normal. Exclusion criteria were as follows: 1 Patients had the history of dislocation or subluxation of patella or excessive Q angle (> 20°); 2 Severe patellofemoral arthritis was not suitable for LPRR alone; 3 Other diseases were not suitable for surgery treatment.

Sixty six cases (23 males and 43 females) were included in the TSM group, aged 16–65 years with an average age of 48.3 years. Thirty-two cases were in the left knees and 34 cases in the right knees. The medical history was 6–29 months, with an average of 18.5 months. Thirty cases had traumatic history. Arthroscopic articular cartilage injury was assessed according to outerbridge classification [11]. Grade I-II was in 51 cases, grade III in 10 cases, and grade IV in 5 cases. Twenty one cases had meniscus injury, 10 cases synovial fold hyperplasia and 8 cases free body (Table 1).

**Table 1:** Basic characteristics of patients

Parameter	TSM group	OSM group
Cases	66	59
Gender		
Men	23	18
Women	43	41
Age (Year)	16–65 (48.3)	17–66 (49.2)
Knee		
Left knee	32	28
Right knee	34	31
Medical history(M)	6–29 (18.5)	5–29 (19.1)
History of trauma (Cases)	30	27
Outerbridge rating		
I-II	51	45
III	10	8
IV	5	6
Combined meniscus injury	21	20
Synovial fold hyperplasia	10	8
Free body	8	8

Fifty-nine cases were included in the OSM group, including 18 males and 41 females. The age ranged from 17 to 66 years, with an average age of 49.2 years. Twenty-eight cases were in the left knees and 31 cases in the right knees. The medical history was 7–29 months, with an average of 19.1 months. Twenty-seven cases had traumatic history. Outbridge classifications were as follows: Grade I-II was in 45 knee, III 8 knee, and IV 6 knee. Twenty cases had meniscus injury, 8 cases synovial fold hyperplasia and 8 cases free body (Table 1).

### Surgical techniques

The patients were placed supine, and tourniquet was tied at the base of thigh. The affected limb drooped at the bedside or at the end of bed after continuous epidural anesthesia. Conventional anterolateral portal (AL), anteromedial (AM), superolateral portal (SL) and superomedial portal (SM) were used for surgery. The AL approach was used for comprehensive examination of joint cavity, and then used to conduct patelloplasty of the degenerated articular surface, clean the hypertrophic inflammatory synovial tissue and synovial fold of patellofemoral joint, trim the ruptured meniscus and remove the free body and unstable cartilage fragments. The relationship between patella and lateral femoral condyle and the cartilage changes of lateral patella articular surface were observed during the flexion and extension of knee joint to determine the location and extend of LPRR. The TSM group entered the release knife through the AL approach, and the AM or SM approach under arthroscopy were used to monitor the cut process of synovial membrane, joint capsule and LPR layer by layer at 1 cm from the outer and upper margin of the patella. LPRR was cut longitudinally from proximal end to distal end. Superficial fascia was cut to subcutaneous tissue until the patella returned to its normal position. Satisfactory release should allow the patella to move more than 12 mm inward and tilt the patella to 90 degrees. The range of the release usually includes the shallow and deep layer of LPR and the partial fibrous joint capsules, the part of lateral femoral muscles and the partial structure of iliotibial band patella attachment point. To prevent postoperative bleeding, a dedicated gasification ablation electrocautery was used to cut and stop bleeding. The surgeon flushed joint cavity, pressed the lateral edge of the patella with a small piece of gauze, and then pressed elastic bandage to slightly displace medial side of the patella. In the OSM group, the surface of LPR was bluntly separated through the SL and AL approaches to form a cavity for arthroscopic operation on the surface of LPR. The electric burner was used to remove LPR, the partial fibrous joint capsules, the partial lateral femoral muscles and the partial structure of iliotibial band patella attachment point. Synovial membrane was retained. The range of release extends were down to the tibial tuberosity and up to the tendon junction of lateral femur and rectus femoris. The drainage tube was placed at the release point, and there was no need to place drainage in the joint cavity.

### Postoperative management

The drainage tube was removed at 24-48 h after surgery. Quadriceps muscle strength training, patella medial pushing training and progressive knee joint activity training were started under the support protection at 48 h after surgery. The knee joint activity was controlled within 0–90 degrees within 2 weeks after the operation. The knee joint activity increased to 0–120 degrees within 3–4 weeks. After 4 weeks, comprehensive activity training and muscle strength training were started, and normal activity was resumed after the fully restoration of range of motion and muscle strength.

### Evaluation of clinical curative effect

Each patient was followed up by telephone or outpatient visit every month after surgery and every 3 months after 3 months after surgery. Lyshohn score (excellent: greater than 90 points, good: 80–90 points, better: 70–79 points, poor: less than 70 points), medial shift of patella, Kujala score, VAS, and surgical complications were used and evaluated for efficacy evaluation [6, 12–15].

### Statistical analysis

All statistical analyses were performed using SPSS 13.0 statistical software (SPSS, Chicago, IL, USA). Measurement data were expressed as mean ± standard deviation, and t test and paired z test were used for statistical analysis. The frequency of complications was analyzed using Chi Square with Yates Correction and the Fisher’s Exact test. G*Power 3.1.9.2 software (http://www.gpower.hhu.de/) was used for statistical power analysis. *P* ≤ 0.05 was considered statistically significant.

## Results

In the TSM group, all patients were followed up for 1.5–5 years, with an average of 3.4 years. Sixty-six patients had pain when going up and down stairs and squatting down before surgery. Anterior knee pain was significantly reduced or disappeared in all patients 1 month after surgery. In 1 year after surgery, 55 (83.3%) of the patients were able to go up and down the stairs normally and 11 cases had pain when going up and down stairs and the function was slightly limited. 51 cases of anterior knee pain were basically disappeared at 1 year after surgery, and 15 cases had occasional pain. Lysholm scores were as follows: 44 cases were excellent, 12 cases good, 7 cases better, and 3 cases poor. The effect of LPRR alone in 3 cases of severely damaged articular cartilage was poor, but the pain was relieved to some degree. The excellent rate was 84.8%. Five patients had haemarthrosis in the postoperative period, and the symptomatic treatment such as puncture and drainage was improved. Three cases of joint adhesion were relieved after manual loosening. Infection, deep vein thrombosis and other complications were not observed. The Lysholm scores were 77.4 ± 2.7 before surgery and 94.3 ± 3.4 at the last follow-up, and the difference was statistically significant (*P* < 0.05). The medial pushing distances of patella were 0.76 ± 0.21 cm before operation and 1.25 ± 0.27 cm at the last follow-up (*P* < 0.001). The Kujala scores were 60.4 ± 8.6 before surgery and 84.5 ± 9.2 points at the last follow-up (*P* < 0.00 l). The VAS were 7.5 ± 2.5 before operation and 2.3 ± 1.3 at the final follow-up (*P* < 0.001) (Table 2).

**Table 2:** Comparison of parameters of patients before surgery and at the last follow-up

Parameter	TSM	The last follow-up	Power(1-β err prob)	OSM	The last follow-up	Power(1-β err prob)
	Before surgery			Before surgery		
Cases		66			59	
Follow-up time (Year)		1.5–5 (3.4)			1.5–5 (3.5)	
Lysholm rating						
Excellent		44			43	
Good		12			8	
Better		7			6	
Poor		3			2	
The excellent rate		84.8%			86.4%	
Postoperative Complications					#	0.82
None		58			58	
Hemarthrosis		5			0	
Synarthrophysis		3			1	
Lysholm score	77.4 ± 2.7	94.3 ± 3.4*	1	76.7 ± 2.8	95.4 ± 3.5*	1
Medial displacement of patella	0.76 ± 0.21	1.25 ± 0.27*	1	0.78 ± 0.23	1.28 ± 0.19*	1
Kujala score	60.4 ± 8.6	84.5 ± 9.2*	1	60.7 ± 8.7	84.2 ± 8.9*	1
VAS	7.5 ± 2.5	2.3 ± 1.3*	1	7.4 ± 2.4	2.1 ± 1.2*	1

* Compared with pre-operation, the last follow-up *P* < 0.001

# Compared with TSM, the last follow-up *P* < 0.05

**Fig. 2:**
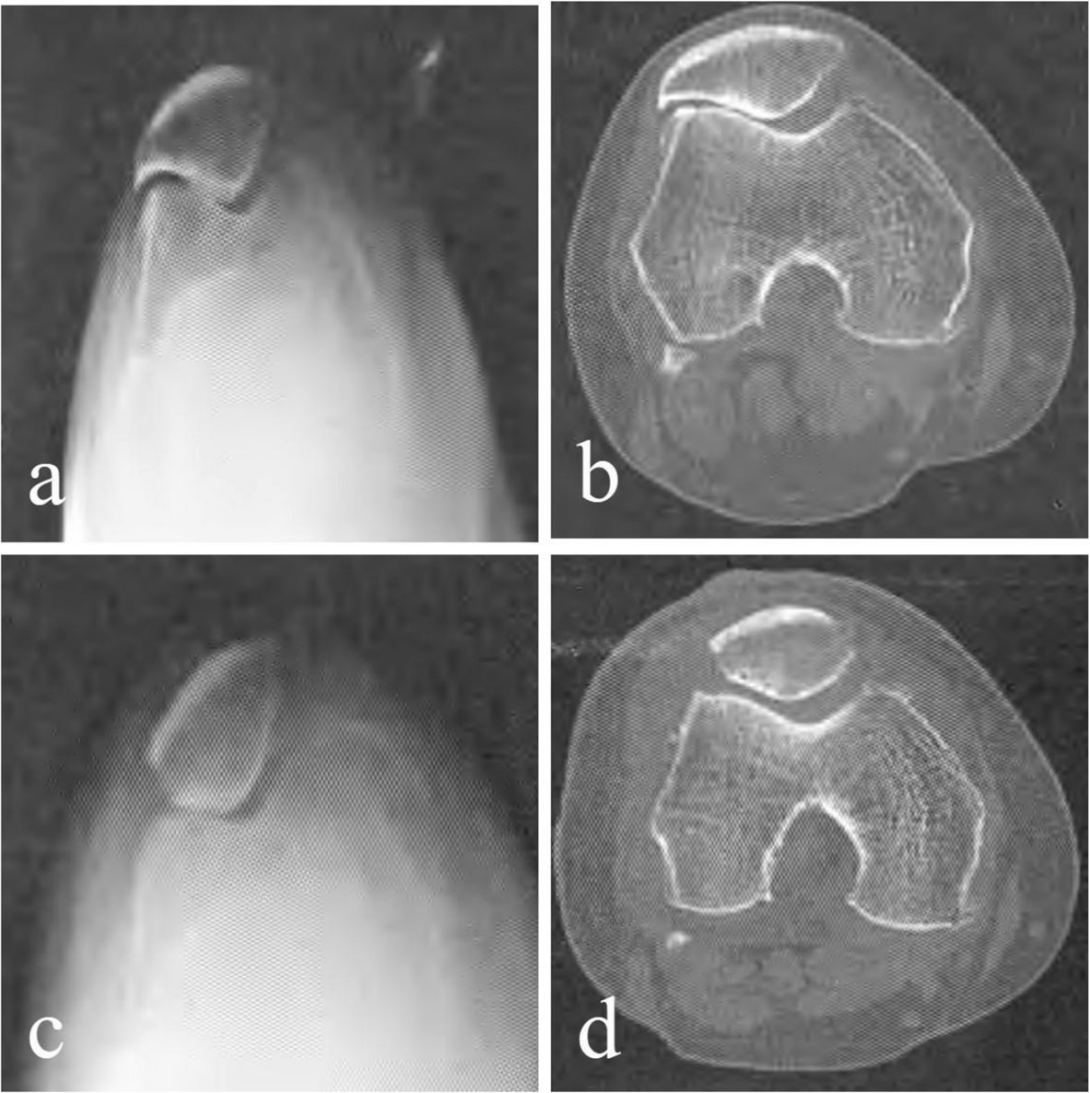
Comparison of preoperative and postoperative radiographic data of X-ray and CT examination of the ELPS patient. **a**, **b** Preoperative tangential X-ray imaging and CT tomography showed hook-shaped patella; the lateral space of the patellofemoral joint was significantly narrow and osteophyte formation was obvious. **c**, **d** Postoperative tangential X-ray imaging and CT tomography showed significant widening of the lateral space of the patellofemoral joint and its medial and lateral space restored to balance; the lateral hook-shaped patella had been shaped into a V-shaped patella; the osteophyte around the lateral patellofemoral joint had been removed and the joint returned to normal

All cases in the OSM group were followed up for 1.5–5 years with an average of 3.5 years (Fig. 2). Fifty-nine patients had pain when going up and down stairs and squatting down before surgery. Anterior knee pain was significantly reduced or disappeared in all patients 1 month after surgery. In the first year after surgery, 50 (84.7%) cases were able to go up and down stairs normally, and 9 cases had pain when going up and down stairs and its function was slightly limited. Fifty cases of anterior knee pain were basically disappeared at 1 year after surgery, and 9 cases had occasional pain. Lysholm scores had excellent in 43 cases, good in 8 cases, better in 6 cases, and poor in 2 cases. The effect of LPRR alone in 2 cases of severely damaged articular cartilage was poor, but the pain was lower than before. The excellent rate was 86.4%. Postoperative complications such as infection, joint adhesion, joint hematoma, and deep vein thrombosis were not reported. The Lysholm scores were 76.7 ± 2.8 before surgery and 95.4 ± 3.5 at the last follow-up, and its difference was statistically significant (*P* < 0.05). The medial pushing distances of patella were 0.78 ± 0.23 cm before operation and 1.28 ± 0.19 cm at the last follow-up (*P* < 0.001). The Kujala scores were 60.7 ± 8.7 at preoperation and 84.2 ± 8.9 at the final follow-up (*P* < 0.001). The VAS scores were 7.4 ± 2.4 before surgery and 2.1 ± 1.2 points at the final follow-up (*P* < 0.001) (Table 2).

There were no significant differences of Lysholm rating and scoring, medial pushing distance of patella, Kujala score, and VAS before surgery and at the last follow-up between the OSM group and TSM group (*P* > 0.05, respectively). However, the number of occurrences of joint hematoma and adhesion was significantly higher in the TSM group than the OSM group (*P* = 0.024, Table 2).

## Discussion

All cases in this study were followed up for 1.5–5 years. All patients had significant reduction or disappearance of anterior knee pain at 1 month and 1 year after surgery. The Lysholm score, the medial pushing distance of patella, Kujala score, and VAS in the OSM group and the TSM group were significantly improved at the last follow-up compared with before surgery(*P* < 0.05). Five patients had heamarthrosis postoperatively in the TSM group, and comprehensive treatments such as rest, local compression bandaging, ice compress, raising the affected limb, puncture and drainage of joint cavity, and plaster or brace braking were managed for those patients. All the patients were all relieved and returned to normal. After the symptoms were relieved, functional exercise was restored. Three cases of joint adhesion in the TSM group and 1 cases in the OSM group were relieved after manual loosening. Infection, deep vein thrombosis and other complications were not observed in the TSM group. Postoperative complications such as infection, joint hematoma, and deep vein thrombosis were not reported in the OSM group. Arthroscopic closing LPRR for the treatment of LPCS can effectively improve the function and symptoms of patellofemoral joint with the advantages of small trauma, rapid recovery and less complications. But, the number of hemarthrosis and joint adhesion were significantly higher in the TSM group than in the OSM group.

Under normal circumstances, the commissure relationship between patellofemoral joint has good adaptability. When the normal anatomical alignment of patellofemoral joint was disturbed due to various reasons, the muscle strength around patella will be unbalanced and the LPR will be contracted, resulting in abnormal patella trajectory and abnormal contact of the patellofemoral joint surface during knee flexion and extension. The stress in local articular surface of patella will be increased and the inner and outer pressure distribution will be uneven, which causes damage to secondary articular cartilage [1]. As time goes on, cartilage damage will be aggravated and pain in the knee will be produced, which develops into LPCS. Arthroscopy could directly measure cartilage contact pressure during surgery to guide the precise release of LPR, which can balance the stress inside and outside of patellofemoral joint to reduce further cartilage degradation. In this study, there were 30 and 27 cases of traumatic history in the TSM group and the OSM group respectively. So trauma was an important cause of LPCS.

Researchers first proposed that the LPR should be released for the cause of this disorder, so that the laterally displaced patella returns to its normal position, and the pressure between the patellofemoral articular cartilage tends to be balanced [16]. The release method includes three types: incision, arthroscopic assisted incision, and arthroscopic closure releasing [14, 17]. The first two methods need to open patellofemoral joint, and its wound is large. Although it can directly look at the site of LPRR, it cannot directly look at the improvement of the relationship between patellofemoral joint during the release process, and often causes insufficient or excessive release to affect the prognosis.

Closed release LPR under arthroscopy could directly observe the change of patella position, adjust the location and extent of the release, and not need to suture locally [18]. After the surgery, the elastic bandage was pressure-wrapped and fixed in the slightly medial displacement of the patella, which can prevent the formation of blood and hemarthrosis. Besides, early active rehabilitation training for patients after LPRR will significantly improve efficacy and prevent postoperative re-adhesion. Patients in the TSM group and the OSM group were followed up for an average of 3.4 and 3.5 years without serious complications. The position of the patella was basically normal. 3 and 2 cases respectively in the TSM group and the OSM group with severely damaged articular cartilage were not working well. So, patients with Outerbridge grade IV of patella are not suitable for arthroscopic LPRR treatment alone.

Fulkerson et al. [19] found that LPRR can effectively correct the lateral petalla tilt by CT scan before and after surgery. In the TSM group, the synovium, joint capsule and LPR were cut apart under arthroscopy from the joint, resulting in partial synovial membrane loss, which easily caused intra-articular hemorrhage and joint adhesion that affect the surgical effect. The biggest feature of the OSM group was to retain the synovial membrane, which can reduce joint hemorrhage and postoperative adhesion due to the intact synovial membrane. Therefore, we found that the incidences of joint hemorrhage and joint adhesion were significantly higher in the TSM group than the OSM group.

The advantages of closed LPRR under arthroscopy are as follows: ① Small trauma is good for quick recovery, rehabilitation and functional exercise. Besides, arthroscopic debridement can be performed under arthroscopy, which can remove the articular cartilage debris, inflammatory factors and calcium salt crystals that cause pain of knee joint, and remove the swelling and degeneration of articular cartilage, teared meniscus, hyperplastic synovial folds and osteophyte, etc. to improve the internal environment of the joint. ② Patelloplasty could be performed simultaneously to reduce the impact between the lateral articular surface of patellofemoral joint. ③ Arthroscopic closed LPRR can dynamically and intuitively observe patella trajectory, patellofemoral joint contact pressure and the degree of cartilage degeneration of the affected articular surface. Besides, it also can accurately determine the condition and dynamically observe the release effect in time being beneficial to accurate operation.

The surgeons should pay attention to the following points during the operation. (1) The obvious hyperplasia and deformation of lateral patella should conduct patelloplasty. (2) The osteophyte of patella trajectory should be removed; (3) Hemostasis using arthroscopic electrocoagulation needs to be thorough to reduce the risk of blood in the joint and accelerate postoperative recovery. (4) The scope of release should be thorough. Most scholars recommend starting from l-2 cm of the proximal end of patella, at least to the anterio-lateral entrance. Marumoto et al. [20] found that retinacula cut from the inferior third of vastus lateralis tendon down to tibial tubercle.

The shortcomings of this study are the small number of cases. In addition, this was not a randomized controlled trial (RCT); the clinicians and patients were not totally blinded to the group assignment. All of these observations need further confirmation in large-sample multi-center prospective randomized controlled trials.

## Conclusion

Arthroscopic closing LPRR for the treatment of LPCS can effectively improve the function and symptoms of patellofemoral joint with the advantages of small trauma, rapid recovery and less complications. But, the number of occurrences of hemarthrosis and joint adhesion in the TSM group were significantly higher than those in the OSM group.

## Data Availability

The datasets used and/or analysed during the current study are available from the corresponding author on reasonable request.

## Abbreviations

AL: Anterolateral portal
AM: Anteromedial
LPCS: Lateral patellar compression syndrome
LPR: Lateral patella retinaculum
LPRR: Lateral patella retinaculum releasing
OSM: Outside synovial membrane
SL: Superolateral portal
SM: superomedial portal
TSM: Through synovial membrane
